# Extracellular signal-regulated kinase-dependent phosphorylation of histone H3 serine 10 is involved in the pathogenesis of traumatic brain injury

**DOI:** 10.3389/fnmol.2022.828567

**Published:** 2022-09-29

**Authors:** Yu Zhang, Xin Yang, Xinran Hou, Wen Zhou, Changlong Bi, Zhuanyi Yang, Sining Lu, Zijin Ding, Zhuofeng Ding, Yu Zou, Qulian Guo, Michael K. E. Schäfer, Changsheng Huang

**Affiliations:** ^1^Department of Anesthesiology, Xiangya Hospital Central South University, Changsha, China; ^2^Department of Neurosurgery, Xiangya Hospital Central South University, Changsha, China; ^3^Medical College of Xiangya, Central South University, Changsha, China; ^4^National Clinical Research Center for Geriatric Disorders, Xiangya Hospital Central South University, Changsha, China; ^5^Department of Anesthesiology, University Medical Center, Johannes Gutenberg University Mainz, Mainz, Germany; ^6^Focus Program Translational Neurosciences and Research Center of Immunotherapy of the Johannes Gutenberg University Mainz, Mainz, Germany

**Keywords:** ERK, phosphorylation, traumatic brain injury, histone modification, H3S10

## Abstract

Traumatic brain injury (TBI) induces a series of epigenetic changes in brain tissue, among which histone modifications are associated with the deterioration of TBI. In this study, we explored the role of histone H3 modifications in a weight-drop model of TBI in rats. Screening for various histone modifications, immunoblot analyses revealed that the phosphorylation of histone H3 serine 10 (p-H3S10) was significantly upregulated after TBI in the brain tissue surrounding the injury site. A similar posttraumatic regulation was observed for phosphorylated extracellular signal-regulated kinase (p-ERK), which is known to phosphorylate H3S10. In support of the hypothesis that ERK-mediated phosphorylation of H3S10 contributes to TBI pathogenesis, double immunofluorescence staining of brain sections showed high levels and colocalization of p-H3S10 and p-ERK predominantly in neurons surrounding the injury site. To test the hypothesis that inhibition of ERK-H3S10 signaling ameliorates TBI pathogenesis, the mitogen-activated protein kinase–extracellular signal-regulated kinase kinase (MEK) 1/2 inhibitor U0126, which inhibits ERK phosphorylation, was administered into the right lateral ventricle of TBI male and female rats via intracerebroventricular cannulation for 7 days post trauma. U0126 administration indeed prevented H3S10 phosphorylation and improved motor function recovery and cognitive function compared to vehicle treatment. In agreement with our findings in the rat model of TBI, immunoblot and double immunofluorescence analyses of brain tissue specimens from patients with TBI demonstrated high levels and colocalization of p-H3S10 and p-ERK as compared to control specimens from non-injured individuals. In conclusion, our findings indicate that phosphorylation-dependent activation of ERK-H3S10 signaling participates in the pathogenesis of TBI and can be targeted by pharmacological approaches.

## Introduction

Approximately 69 million people worldwide suffer from traumatic brain injury (TBI) every year (Dewan et al., 2018). TBI, with an age-standardized incidence rate of 369 per 100,000 population, causes 8.1 million years of life with disability and imposes heavy economic burdens to health systems worldwide (Maas et al., 2017; James et al., 2019). After the primary injury caused by the initial damage, the secondary injury results from ischemia, edema, inflammation and intracranial hematoma and could progress and lead to deterioration of TBI (Johnson et al., 2018; Morganti-Kossmann et al., 2019). Thus, effective prevention of secondary injury might be a promising way to improve the prognosis of TBI patients.

The pathological mechanisms of secondary injury comprise complex multifactorial processes including oxidative stress, excitotoxicity, neuronal apoptosis, etc. (Kaur and Sharma, 2018). Epigenetic mechanisms, especially posttranslational histone modifications, have been shown to participate in the pathogenesis of TBI (Wong and Langley, 2016; Nagalakshmi et al., 2018; Bertogliat et al., 2020). Histone modifications occurring at multiple sites in multiple forms, such as methylation, acetylation, ubiquitylation, sumoylation, and phosphorylation, can lead to alterations in chromatin structure, accessibility of transcription factor binding sites and subsequent gene expression (Stillman, 2018). Histone H3 acetylation and methylation are increased in the hippocampal CA3 region of immature rats after TBI, indicating altered epigenetic signaling (Gao et al., 2006). Moreover, targeting specific histone modifications or relevant enzymes after TBI may have beneficial effects on different pathophysiological processes. For example, HDAC inhibitors have been shown to reduce the microglial inflammatory response following TBI (Zhang et al., 2008; Lu et al., 2013). HDAC inhibitors also preserve nerve growth factor mediated cell survival and exert neuroprotective effects following TBI in rodent models (Zhang et al., 2008; Lu et al., 2013).

In the present study, we aimed to investigate the role of histone modifications in the pathogenesis of TBI using a rat weight-drop model. We examined the changes in different histone modifications, including H3ac, H3K27ac, H3K4me1, H3K9me3, H3K27me3, and p-H3S10, by western blot analysis. After identifying marked upregulation of p-H3S10, we performed protein expression analyses and examined its coregulation and colocalization with the p-ERK, which is known to phosphorylate H3S10 (Tochiki et al., 2016). We further tested whether pharmacological inhibition of p-ERK/p-H3S10 signaling has a therapeutic benefit after TBI. Finally, the regulation of p-ERK and p-H3S10 was also studied in brain tissue specimens from TBI patients.

## Materials and methods

### Experimental animals

Adult Sprague-Dawley rats (Hunan SLAC Laboratory Animal Co., Ltd., Changsha, China), 7–8 weeks old, were used for all experimental procedures. The animals were group-housed in clear plastic cages with sawdust bedding in a specific pathogen-free room with a temperature of 24–25°C, humidity of 60%, a light/dark cycle of 12/12 h and free access to food and water in the Experimental Animal Center of Central South University (Changsha, Hunan, China). The rats were allowed to habituate to the environment for 4 days prior to experimental procedures, and behavioral tests were performed by an experimenter blinded to the animal groups. All procedures were approved by the Institutional Ethics Committee of Central South University and were carried out in accordance with the National Institutes of Health Guide for the Care and Use of Laboratory Animals.

Experiments with male and female rats were performed. However, initial experiments to validate the TBI model were performed with male rats only and were divided into two parts. The first part comprised three groups of male rats: sham operation group (*n* = 6), 40 g falling weight group (*n* = 6), and 80 g falling weight group (*n* = 6). The second part comprised four groups of male rats including drug administration: sham + vehicle group (*n* = 8), sham + U0126 group (*n* = 8), TBI + vehicle group (*n* = 8), and TBI + U0126 group (*n* = 8).

Finally, female rats were examined in four groups: sham + vehicle group (*n* = 8), sham + U0126 group (*n* = 8), TBI + vehicle group (*n* = 8), TBI + U0126 group (*n* = 8). All animals were subjected to TBI/sham surgery, rotarod test and neurological severity score assessment and molecular biology experiments. In addition, male rats and female rats (*n* = 6, per group each) assigned to either sham + vehicle group (*n* = 6), sham + U0126 group (*n* = 6), TBI + vehicle group (*n* = 6), or TBI + U0126 group (*n* = 6) and were subjected to the Morris water maze test.

### Patient brain tissue specimens

The study was approved by the Ethics Committee of Xiangya Hospital of Central South University (approval number: 201712841) and was registered in the Chinese Clinical Trial Registry^1^ with the registration number ChiCTR1800015070. Written informed consent was obtained from all included patients or their close relatives, and the study was in compliance with the guidelines approved by Xiangya Hospital of Central South University.

The medical data of all participants were reviewed for the inclusion/exclusion criteria. The inclusion criteria were as follows: (1) voluntary participants older than 18 years of age; and (2) brain trauma patients with clear indications for craniotomy surgery who were fully informed and provided informed consent. The exclusion criteria were as follows: (1) patients with psychiatric disorders; (2) pre-existing history of cancer; (3) pre-existing history of systematic infection; and (4) multiple severe injuries associated with other organs. The GCS (Teasdale and Jennett, 1974) was used by two independent neurological surgeons to evaluate each patient’s clinical condition and severity. The damaged frontal lobe cortex tissue was partially removed during the surgery for treatment and collected by snap freezing. In the control group, normal frontal lobe cortex tissue was obtained in the patients who received epilepsy surgery. For these patients, the surgeon had to remove healthy brain tissue in order to get to the epileptic foci (Balan et al., 2013).

### Traumatic brain injury modeling

The focal traumatic brain injury rat model was established using the modified weight-drop method as described (Feeney et al., 1981). Briefly, after anesthesia with 4% isoflurane in 60% air/40% O_2_ for 5 min, rats were placed in a prone position with their heads fixed in a stereotaxic frame (RWD Life Science Co., Ltd., Shenzhen, China). During the surgery, anesthesia was maintained with 2–2.5% isoflurane. A longitudinal midline scalp incision was made to expose the bony skull, and a metal circular tip with diameter 1.5 mm was placed upon the surface of dura. The hit site was located 0.5 mm posterior to bregma and 3.0 mm right lateral to the sagittal suture (Smith et al., 2007). Falling weights with an impact velocity of either 40 or 80 g (released from a height of 20 cm) and a deformation depth below the dura of 2 mm were used with a weight-drop hitting device (RWD Life Science Co., Ltd., Shenzhen, China). For the sham-operated group, rats were subjected to the same procedure but without a weight drop. Then, the incision was sutured routinely, and the rats were returned to their home cages to recover.

### Intracerebroventricular cannulation and drug administration

An intracerebroventricular cannula was implanted into the right lateral ventricle for drug delivery 7 days before TBI. The procedure was performed as described (Jho et al., 2003) with slight modifications. In brief, the rats were anesthetized, and the scalp was incised longitudinally; then, subcutaneous tissue was bluntly dissected to expose the skull. A 0.5-mm hole was drilled using the following flat skull coordinates: 0.9 mm posterior to bregma and 1.5 mm right lateral to the midline (Smith et al., 2007). Then, a steel cannula (ID 0.34 mm/OD 0.48 mm) was implanted 4.5 mm ventral to the surface of the skull, with clear spontaneous cerebrospinal fluid outflow confirming the location. Through methylene blue injection, we ensured the correct placement of the cannula and the intraventricular injection of the drug (Supplementary Figure S1). After the cannula was fixed with dental cement, the incision was sutured as described above. During drug administration, a corresponding inner catheter (ID 0.14 mm/OD 0.30 mm) connected to a microsyringe was placed into the cannula; after drug administration was finished, the inner catheter was removed, and the cannula was capped.

### Drug administration

U0126 (1,4-diamino-2,3-dicyano-1,4-bis[2-aminophenylthio]butadiene) (Sigma-Aldrich, St Louis, MO, United States), an inhibitor of the MEK 1/2, was dissolved in 10% DMSO and 50% PEG300 at 4 μg/5 μl (1.25 μg/μl) and intracerebroventricularly injected at a dose used in previous studies (Bharne et al., 2016; He et al., 2018; Jiang et al., 2018; Somalwar et al., 2018). The injection of U0126 or vehicle was performed once daily from immediately after injury to the seventh day after injury.

### Behavioral tests

#### Rotarod test

The rotarod test was performed on the day before injury and on the first, third, fifth, and seventh days after injury to evaluate motor function. The rotarod treadmill device (Shanghai Biowill Co., Ltd., China) consisted of a base platform and, placed at a height of 15 cm above the base, a horizontal rotating rod (3 cm diameter, 30 cm length) with a non-slip surface divided into five equal sections by six disks. The rotation speed was set to accelerate from 0 to 30 rpm in 10 s. The latency of rats to fall from the rotating rod was recorded (cutoff time was set as 100 s). Each rat was tested three times with 5 min intervals, and the final performance time was averaged.

#### Neurological severity score assessment

Assessment of the NSS was conducted on the day before injury and on the first, third, fifth, and seventh days after injury. The NSS evaluates somatomotor and somatosensory functions by testing the performance of the animals in motor, sensory, reflex, beam walking, and beam balancing tasks. The total scores ranged from 0 to 24, in which a higher score indicated more severe impairment of nervous function. The scoring system was adapted from previously published methods and is shown in Table 1 (Ling et al., 2004).

**TABLE 1:** Neurological severity score grading.

	Score (If unable, score 1; If able, score 0.)
Inability to exit from a circle 50 cm in diameter when placed at the center	
Within 30 min	
Within 60 min	
>60 min	
Loss of righting reflex	
For 20 min	
For 40 min	
For >60 min	
Hemiplegia: inability of the rat to resist forced changes in position	
Flexion of the hindlimb when raised by the tail	
Inability to walk straight	
Inability to move	
Loss of the startle reflex	
Loss of the pinna reflex	
Loss of seeking behavior	
Prostration	
Loss of placing reflexes	
Right forelimb	
Left forelimb	
Right hindlimb	
Left hindlimb	
Balance beam (1.5 cm wide)	
<20 s	
<40 s	
<60 s	
Beam walking	
Failure on a 2.5 cm wide beam	
Failure on a 5.0 cm wide beam	
Failure on an 8.0 cm wide beam	

#### Morris water maze test

Morris water maze (MWM) test was performed to measure cognitive function of rats after TBI according to previous studies (Tucker et al., 2018). Briefly, a platform of 12 cm in diameter was placed in a cylindrical tank (150 cm in diameter) filled with water (22°C, 30 cm in depth) to 2 cm over the top surface of the platform. A cross in the bottom center of the tank divided the waters equally into four quadrants, and the platform was placed in the center of one quadrant. On the training days, rats were put into the water from four quadrants, respectively, and was allowed to swim freely in the maze until they found the platform within 120 s. Each rat was trained daily from the third day to the sixth day after TBI. On the seventh day after TBI, the platform was removed and the probe test was performed. Smart 3.0 system (Panlab) was used to track and analyze behavioral parameters.

### Western blot

Rats were deeply anesthetized and transcardially perfused with 300 ml 0.1 M PBS. Then, the right lateral cerebral cortex was collected on ice according to the following brain coordinates: bregma 5.16 mm, interaural 4.02 mm to bregma −3.24 mm, interaural 1.98 mm (Watson, 2014) and snap frozen in liquid nitrogen. Tissue specimens were homogenized in RIPA lysis buffer (Beyotime, Shanghai, China) with protease inhibitor and phosphatase inhibitor cocktail, followed by centrifugation at 14,000 rpm for 30 min at 4°C. Then, the supernatant was collected, and the protein concentration was measured by a BCA Protein Assay Kit (Beyotime, Shanghai, China). After loading buffer was added, the samples were denatured by heating at 99°C for 5 min. Lysates (50 μg per lane) were separated by standard gel electrophoresis on a 12% SDS-PAGE gel, transferred to Immun-Blot PVDF membranes (Millipore, United States) and blocked with 5% bovine serum albumin for 2 h at room temperature. Then, the membranes were incubated with the following primary antibodies: rabbit anti-GAPDH (1:2000, Abcam, United Kingdom), rabbit anti-p-H3S10 (1:500, Abcam, United Kingdom), rabbit anti-p-ERK (1:1000, CST, United States), mouse anti-H3K4me1 (1:500, Active Motif, United States), mouse anti-H3K9me3 (1:500, Active Motif, United States), mouse anti-H3K27me3 (1:500, Active Motif, United States), mouse anti-H3K27ac (1:500, Active Motif, United States), mouse anti-H3ac (1:500, Active Motif, United States), and rabbit anti-pan-H3 (1:1000, Wuhan Servicebio Technology Co., China) at 4°C overnight. After the membranes were rinsed with TBST solution, they were incubated with the corresponding horseradish peroxidase (HRP)-conjugated secondary antibodies, goat anti-rabbit or anti-mouse (1:5000), at room temperature for 2 h. After the membranes were rinsed again with TBST, the signal was visualized with ECL substrate and detected by a ChemiDoc XRS System (Bio-Rad, United States) or Amersham Imager 600 (GE, United States), and analyzed using Image Lab Software or ImageJ Software.

### Immunofluorescence staining

Cerebral cortex tissue specimens were harvested as described above, but the perfusion fluid was 300 ml 0.1 M PBS plus 280 ml 4% paraformaldehyde in 0.1 M PBS for prefixation. Then, the dissected specimens were postfixed in 4% paraformaldehyde for another 8 h at 4°C, followed by dehydration in 15% and 30% sucrose successively. After the specimens were embedded in Tissue-Tek^®^ O.C.T. (optimal cutting temperature) compound (Sakura, Japan), serial transverse frozen sections were cut at 10 μm thickness inside a cryotome (Leica, Germany).

After the sections were rinsed with PBS, they were blocked with 5% normal donkey serum in PBS containing 0.3% Triton X-100 for 2 h at room temperature and then incubated with the following primary antibodies: rabbit anti-p-H3S10 (1:200, Abcam, United Kingdom), goat anti-Iba-1 (1:200, Wako, Japan), mouse anti-GFAP (1:400, Dako, Denmark), and mouse anti-NeuN (1:200, Millipore, United States) in a wet box at 4°C overnight. After rinsing three times with PBS, the sections were then incubated with Alexa Fluor 594-labeled donkey anti-goat IgG (1:200, Jackson ImmunoResearch Laboratories, United States), DyLight 594-labeled donkey anti-mouse IgG (1:200, Jackson ImmunoResearch Laboratories, United States) and/or DyLight 488-labeled donkey anti-rabbit IgG (1:200, Jackson ImmunoResearch Laboratories, United States) fluorescent secondary antibodies at room temperature for 2 h. After another three rinses, the sections were coverslipped with mounting medium containing DAPI. Fluorescent images were captured on a Leica DM5000B microscope (Leica, Germany) with identical acquisition parameters among different specimens.

### Statistical analysis

All data are presented as the mean ± SD. Two-way ANOVA with repeated measures followed by Tukey’s multiple comparisons test was performed for the data of rotarod test and NSS. One-way ANOVA followed by Dunnett’s multiple comparisons test or Student’s unpaired *t*-test were used to analyze Morris water maze test data. One-way ANOVA followed by Holm-Šídák multiple comparisons test or the Mann–Whitney test for pairwise-comparisons were used to analyze western blot data. Differences were considered statistically significant at *p* < 0.05. GraphPad Prism software version 8.0.1 (GraphPad Software Inc., United States) was used for all statistical analyses and graphs.

## Results

### Impaired motor function and cognitive function in rats after traumatic brain injury

**FIGURE 1:**
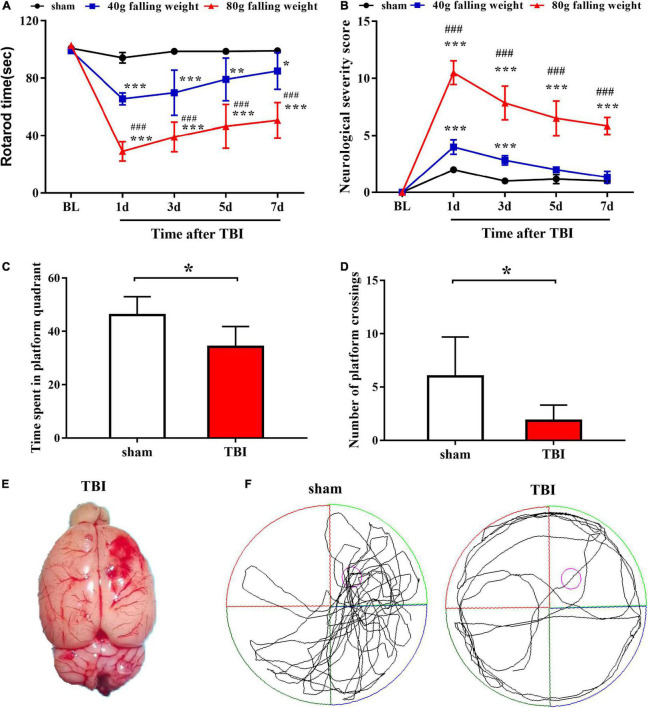
Traumatic brain injury caused significant motor dysfunction and nerve function damage, as tested by the rotarod assay, neurological severity score scale and Morris water maze test. Behavior was evaluated at multiple time points by the rotarod test **(A)** and neurological severity score **(B)**, which indicated persistent damage to motor function in TBI (*n* = 6 rats per group. Two-way ANOVA with repeated measures followed by Tukey’s multiple comparisons test. *p < 0.05, **p < 0.01, ***p < 0.001, sham vs. 40 g falling weight; ^###^*p* < 0.001, 80 g falling weight vs. 40 g falling weight. The values are expressed as the means ± SD. Baseline, BL). The time spent in the platform quadrant **(C)** and the number of platform crossings **(D)** were recorded to assess the cognitive function of rats (*n* = 6 rats per group. Student’s unpaired *t*-test, *p < 0.05. TBI vs. sham. The values are expressed as the means ± SD). **(E)** Photograph of a rat brain showing a typical lesion from TBI over the right frontal lobe. **(F)** The swim paths shown for sham and TBI groups were those of a single animal in the probe test. The hidden platform was indicated by the purple circle.

We constructed a weight-drop TBI model in rats to induce cortical contusion in the frontal lobe of the right brain (Figure 1E). The behavioral tests showed impaired motor function at the first day after TBI followed by gradual recovery until the seventh day after TBI. The rotarod times of the two TBI groups both decreased significantly on the first day after injury, while the sham group showed no significant alterations. The rotarod time of the 40 g falling weight group recovered to approximately 85% of the sham group on the seventh day after injury (85.00 ± 12.76 vs. 99.00 ± 1.67, *p* < 0.05), while the rotarod time of the 80 g falling weight group only recovered to approximately 50% of the sham group (50.56 ± 12.43 vs. 99.00 ± 1.67, *p* < 0.001) (Figure 1A). The NSS of the two TBI groups showed a peak on the first day after injury, indicating serious impairment of motor function. The NSS of the 40 g falling weight group recovered to a level that was not significantly different from that of the sham group on the seventh day after injury (1.33 ± 0.52 vs. 1.00 ± 0.00, *p* > 0.05), while the 80 g falling weight group still had a score of 5.83 ± 0.75 (Figure 1B). The above results showed increased duration of motor impairment in the 80 g falling weight group as compared to the 40 g falling weight group. As shown in Figures 1C,D, the time spent in platform quadrant and the number of platform crossings of TBI rats with the 80 g falling weight were reduced compared with the sham group in the MWM test (*p* < 0.05), suggesting that TBI rats showed no clear preference for the target quadrant, whereas sham rats showed a significant target quadrant preference. The swim paths showed that sham rats gained memory in learning the location of the platform while TBI rats spent more time circling the perimeter of the pool indicating a deficit in spatial memory (Figure 1F). These results suggested that TBI rats developed cognitive impairments.

### Phosphorylation of histone H3 serine 10 and extracellular signal-regulated kinase was upregulated after traumatic brain injury

**FIGURE 2:**
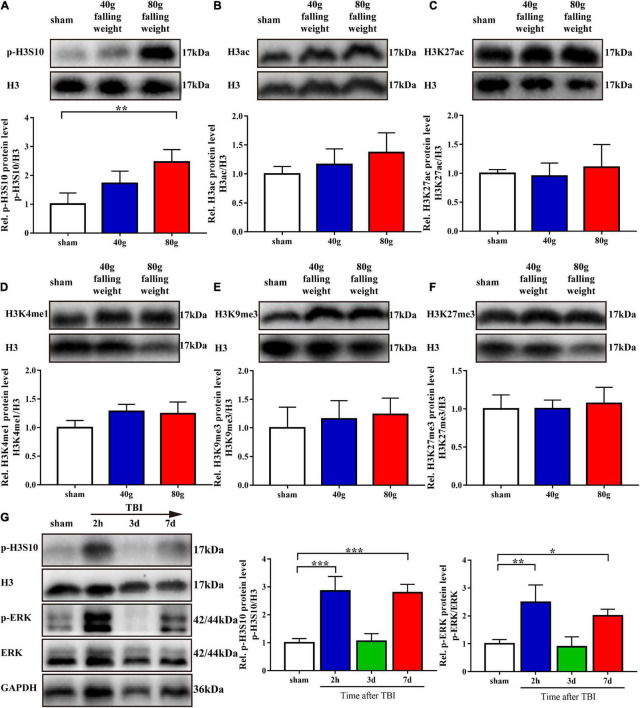
Phosphorylation of histone H3 Ser10 was upregulated in TBI. The level of p-ERK showed variation trends similar to those of p-H3S10 at multiple time points. **(A–F)** Protein bands from western blots for p-H3S10, H3ac, H3K27ac, H3K4me1, H3K9me3, H3K27me3, and H3. The expression of each protein was normalized to that of H3. On the seventh day after TBI, the relative protein level of p-H3S10 showed a significant increase in the TBI rats compared with the sham rats, whereas no change was observed for H3ac, H3K27ac, H3K4me1, H3K9me3, or H3K27me3 (*n* = 4 rats/group. One-way ANOVA followed by Holm-Sidak’s multiple comparisons test. *p < 0.05, **p < 0.01, compared with sham. The values are expressed as the means ± SD). Measurement of p-H3S10 and p-ERK **(G)** levels at multiple time points showed increases at 2 h and on the seventh day after TBI (*n* = 3 rats/group. One-way ANOVA followed by Holm-Sidak’s multiple comparisons test. *p < 0.05, **p < 0.01, ***p < 0.001, compared with sham. The values are expressed as the means ± SD).

We screened for several histone H3 modifications in the brain tissue around the injury site on the seventh day after TBI. Western blot analysis showed that, compared with that in the sham group, only the p-H3S10 level was upregulated in the 80 g falling weight group (*p* < 0.01). The levels of other histone H3 modifications, including H3ac, H3K27ac, H3K4me1, H3K9me3, and H3K27me3, showed no significant changes (Figures 2A–F).

As sustained impairment is observed in clinical TBI, we used the 80 g falling weight TBI model for subsequent experiments. The phosphorylation level of H3S10 after TBI and the identity of its upstream kinase were evaluated. Western blot analysis showed that the level of p-H3S10 was significantly increased at 2 h after injury, returned to the control level on the third day after injury, and increased again on the seventh day after injury (Figure 2G). To identify the upstream kinase of H3S10, we assessed the levels of p-ERK in the ERK pathway and AURKB in the aurora kinase pathway. The p-ERK level was increased at 2 h after injury and on the seventh day after injury, which was similar to the changes observed for the p-H3S10 level (Figure 2G). The AURKB level showed no significant changes (Supplementary Figure S2).

### Phosphorylation of histone H3 serine 10 and phosphorylated extracellular signal-regulated kinase were highly expressed in brain tissue around the injury site and mainly colocalized in neurons

**FIGURE 3:**
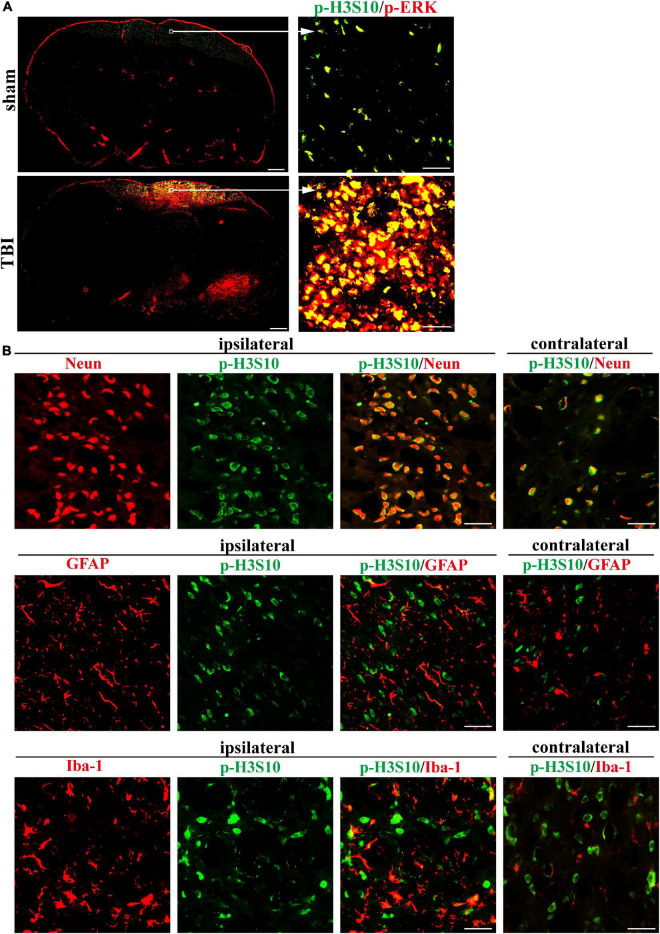
Immunofluorescence in rat brain sections shows high levels of p-H3S10 and p-ERK in the cortex adjacent to the TBI site and their colocalization on the seventh day after TBI. **(A)** Both p-H3S10 and p-ERK were highly expressed in the region near the injury site compared with their levels on the uninjured side. p-H3S10 and p-ERK were colocalized and upregulated in the TBI rats compared with the sham rats. Scale bar = 1000 μm (left) and 50 μm (right), respectively. **(B)** p-H3S10 was colocalized with NeuN (neuron marker), GFAP (astrocyte marker) and Iba-1 (microglia marker) in the TBI rats. Scale bar = 50 μm.

We collected rat brain tissue on the seventh day after TBI and performed immunofluorescence staining to explore the spatial characteristics of p-H3S10 and p-ERK. A panoramic view of a coronal section at the injury site showed the levels and distribution of p-H3S10 (green fluorescence) and p-ERK (red fluorescence) in the brain. Compared with the healthy (left) side, p-H3S10 and p-ERK were highly expressed in the frontal cortex and subcortical brain tissue on the injured (right) side. Compared with those in the sham group, the fluorescence intensities of p-H3S10 and p-ERK in the right frontal lobe area in the TBI group were increased, and p-H3S10 and p-ERK were colocalized (Figure 3A). We observed that the p-H3S10 was predominantly colocalized with NeuN, and colocalized to a minor degree with GFAP and Iba-1 (Figure 3B).

### Treatment with U0126 reversed the upregulation of phosphorylated extracellular signal-regulated kinase and phosphorylation of histone H3 serine 10 and improved behavioral performance in traumatic brain injury rats

**FIGURE 4:**
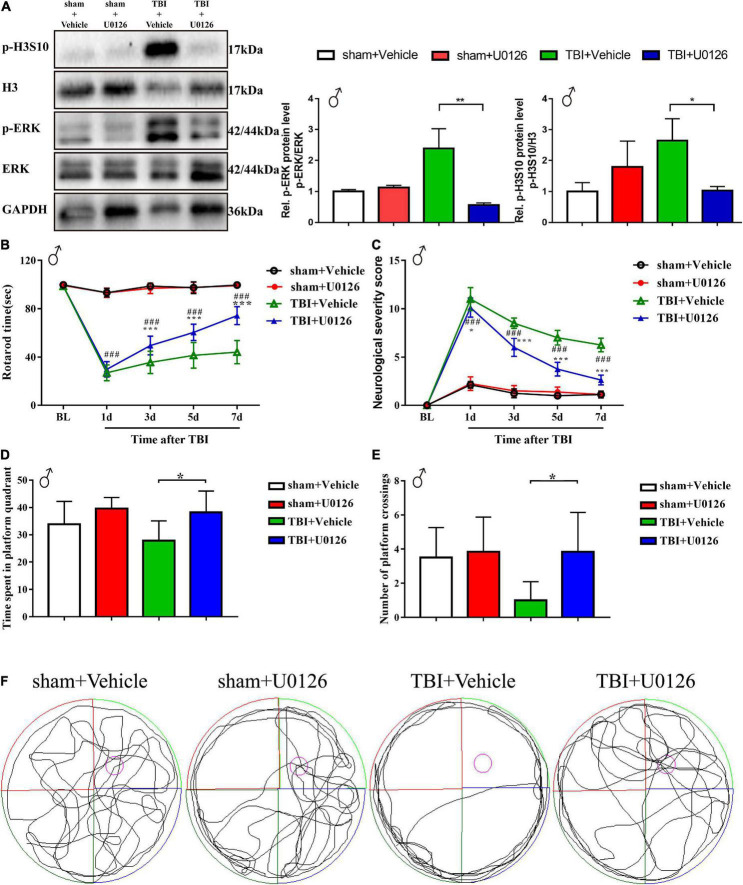
Treatment with U0126, an inhibitor of p-ERK, reversed the upregulation of p-ERK and p-H3S10 in TBI male rats, improved rotarod test and Morris water maze test performance, and attenuated neurological severity score in TBI male rats. Western blot analysis showed that the phosphorylation levels of **(A)** ERK (**p < 0.01) and H3S10 (*p < 0.05) were reversed by U0126 treatment in the TBI rats (*n* = 6 rats/group. Mann–Whitney test. TBI + U0126 vs. TBI + vehicle. The values are expressed as the means ± SD). Motor function was improved between the third and seventh days after injury by U0126 as shown in the rotarod test **(B)** and neurological severity score test **(C)** (*n* = 8 rats/group. Two-way ANOVA with repeated measures followed by Tukey’s multiple comparisons test. *p < 0.05, ***p < 0.001, TBI + U0126 vs. TBI + vehicle; ^###^*p* < 0.001, TBI + U0126 vs. sham + U0126. The values are expressed as the means ± SD). Cognitive function was improved as shown in the Morris water maze test **(D,E)** (*n* = 6 rats/group. One-way ANOVA followed by Dunnett’s multiple comparisons test. *p < 0.05, TBI + U0126 vs. TBI + vehicle. The values are expressed as the means ± SD). **(F)** The swim paths shown for sham + U0126, sham + vehicle, TBI + vehicle and TBI + U0126 groups were those of a single male animal in the probe test. The hidden platform was indicated by the purple circle.

To explore the pathophysiological relevance and druggability of ERK-H3S10 signaling, we injected the MEK1/2 inhibitor U0126 into the lateral ventricle of the injured (right) hemisphere immediately after TBI modeling through a pre-implanted cannula. This inhibitor has been used before in experimental TBI showing various protective effects on neuronal injury, inflammation and edema formation (Mori et al., 2002; Valable et al., 2010). Western blot analysis of the brain tissue around the injury site showed that U0126 treatment significantly decreased the p-ERK level in the TBI group as compared to the vehicle group on the seventh day after the injection of U0126 (*p* < 0.01), while the p-ERK level in the sham group were unchanged (Figure 4A). This result indicated that U0126 successfully suppressed the TBI-induced phosphorylation of ERK. The western blot analysis also revealed that U0126 significantly reduced the increase in p-H3S10 level in the TBI group (*p* < 0.05), while p-H3S10 levels were not significantly affected in the sham group (Figure 4A). The above results suggested that treatment with U0126 suppressed the p-ERK level and suppressed the downstream p-H3S10 level, thus reversing their upregulation in the TBI rats.

Behavioral analyses showed between the third and seventh days after TBI an improved motor function recovery of the TBI rats treated with U0126 compared to vehicle control rats, as evidenced by a longer rotarod time and a lower NSS score (Figures 4B,C). On the seventh day after TBI, the rotarod time of the U0126 group was approximately 1.68 times that of the vehicle control group (74.29 ± 7.46 vs. 44.08 ± 9.60, *p* < 0.001), while the NSS was approximately 0.42 times that of the vehicle control group (2.63 ± 0.52 vs. 6.25 ± 0.71, *p* < 0.001). In the MWM test, TBI rats treated with vehicle showed no preference for the target quadrant indicating a deficit in spatial reference memory. In contrast, U0126 increased the time spent in the platform quadrant and the number of platform crossings in TBI rats (Figures 4D,E, *p* < 0.05). In addition, TBI rats treated with U0126 spent less time circling the perimeter of the pool (Figure 4F). These results suggested that pharmacological inhibition of TBI-induced ERK-H3S10 signaling improved motor and cognitive function after TBI.

**FIGURE 5:**
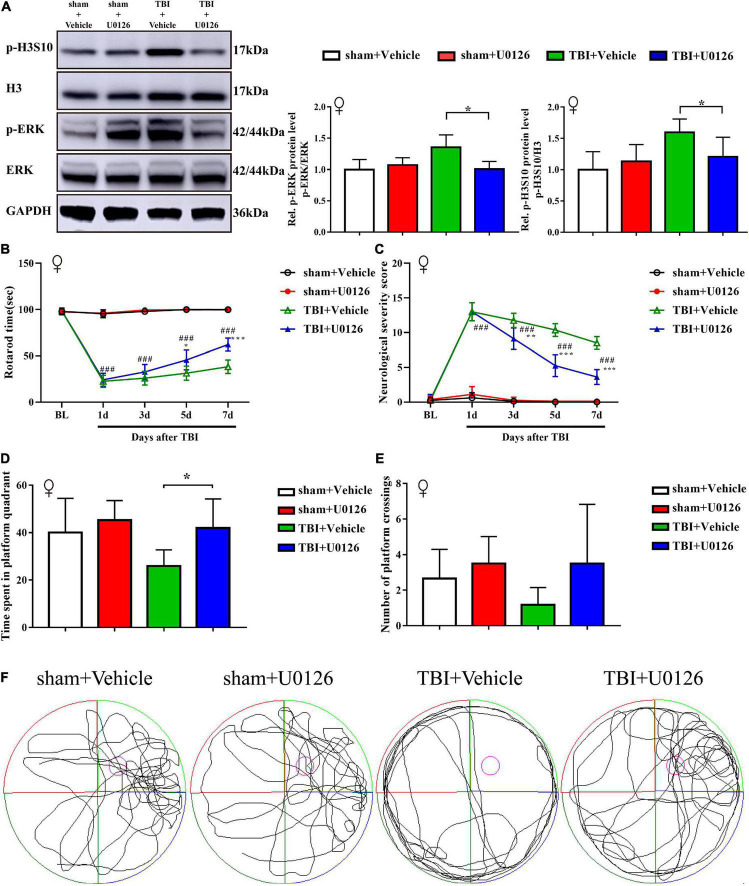
Treatment with U0126 prevented the upregulation of p-ERK and p-H3S10 in female TBI rats, improved rotarod test and Morris water maze test performance, and attenuated neurological severity score in TBI female rats. **(A)** Western blot analysis showed that U0126 treatment reversed the phosphorylation levels of ERK and H3S10 (*p < 0.05) in TBI female rats (*n* = 6 rats/group. Mann–Whitney test. TBI + U0126 vs. TBI + vehicle. The values are expressed as the means ± SD). Motor function was improved between the third and seventh days after injury by U0126 as shown in the rotarod test **(B)** and neurological severity score test **(C)** (*n* = 8 rats/group. Two-way ANOVA with repeated measures followed by Tukey’s multiple comparisons test. *p < 0.05, ***p < 0.001, TBI + U0126 vs. TBI + vehicle; ^###^*p* < 0.001, TBI + U0126 vs. sham + U0126. The values are expressed as the means ± SD). Cognitive function was also improved as shown in the Morris water maze test **(D,E)** (*n* = 6 rats/group. One-way ANOVA followed by Dunnett’s multiple comparisons test. *p < 0.05, TBI + U0126 vs. TBI + vehicle. The values are expressed as the means ± SD). **(F)** The swim paths shown for sham + U0126, sham + vehicle, TBI + vehicle and TBI + U0126 groups were those of a single female animal in the probe test. The hidden platform was indicated by the purple circle.

Considering sex-based differences in outcome following TBI in animals and humans, we also validated the effect of U0126 in TBI female rats. Similar to TBI male rats, treatment with U0126 in female rats reversed the upregulation of p-H3S10 and p-ERK after TBI, improved performance on the rotarod test and MWM test, and reduced NSS (Figures 5A–F).

### Phosphorylation of histone H3 serine 10 and extracellular signal-regulated kinase was also upregulated in clinical specimens from patients with traumatic brain injury

**FIGURE 6:**
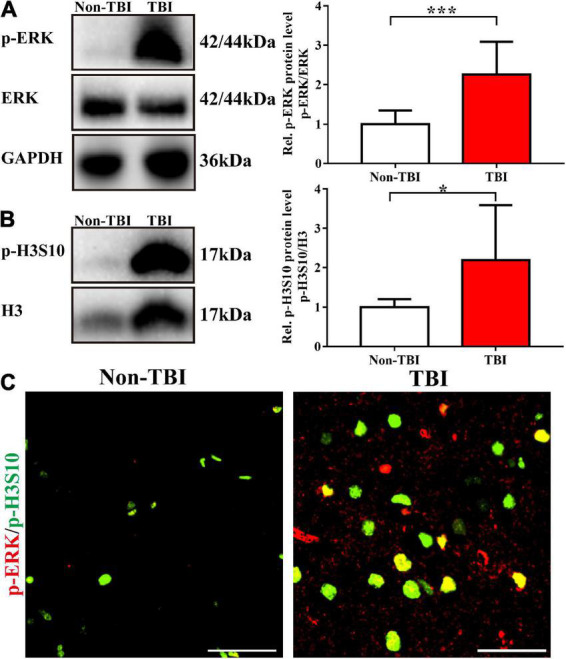
Phosphorylation of H3S10 and ERK was upregulated in clinical brain specimens from patients with TBI. Western blot analysis showed that the levels of p-ERK **(A)** and p-H3S10 **(B)** in the brain tissue of TBI patients were significantly increased compared with those of non-TBI patients (For TBI patients, *n* = 11; for non-TBI patients, *n* = 6. Mann–Whitney test. *p < 0.05, ***p < 0.001. The values are expressed as the means ± SD). **(C)** Immunofluorescence images showing the high levels of p-H3S10 and p-ERK and their colocalization. Scale bar = 50 μm.

We collected brain tissue specimens from 11 TBI patients (TBI group) and 6 epilepsy patients (non-TBI group) during routine surgical procedures after receiving the authorization and consent of the patients or their close relatives and the approval of the Institutional Ethics Committee of Central South University and clinical trial registration. The basic information of the study participants is shown in Table 2. The patients in the TBI group were older than those in the non-TBI group (54.82 ± 14.43 vs. 22.67 ± 3.08, *p* < 0.001), and there was no difference in the sex distribution between the two groups (*p* = 0.620). Western blot analysis showed that the TBI group had higher levels of p-H3S10 and p-ERK than the non-TBI group (Figures 6A,B). Fluorescence immunostaining of brain tissue sections also showed that p-H3S10 levels were higher in TBI patients than in non-TBI patients and that p-H3S10 partially colocalized with p-ERK (Figure 6C). These results suggested that ERK-H3S10 signaling might also be activated in the brain tissue surrounding the injury site in TBI patients.

**TABLE 2:** Clinical data of patients with and without TBI.

	Non-TBI (*n* = 6)	TBI (*n* = 11)	*P*-value
Age (years), mean ± SD	22.67 ± 3.08	54.82 ± 14.43	<0.001
Sex, *n* (%)			0.620
Male	2	6	
Female	4	5	
GCS	14.83 ± 0.41	10.91 ± 3.18	0.010

TBI, traumatic brain injury; GCS, Glasgow Coma Scale.

## Discussion

In this study, we constructed a weight-drop TBI model in rats and found that there was a significant increase in the levels of p-H3S10 and p-ERK in the brain tissue around the TBI injury site. The levels of p-H3S10 and p-ERK exhibited similar changes over time, and immunostaining showed their colocalization predominantly in neurons and to a minor degree in astrocytes and microglia. Administration of the MEK1/2 inhibitor U0126 not only prevented the TBI-induced phosphorylation of ERK and H3S10 but also improved motor and cognitive function recovery within 7 days of injury in male and female rats. In addition, we found high levels of p-H3S10 and p-ERK in brain tissue from TBI patients which suggests that the ERK-H3S10 signaling is critically involved in the pathogenesis of TBI.

Our results are in agreement with earlier findings on histone modifications following brain injury. Histone acetylation and methylation were decreased after controlled cortical impact injury in the hippocampal CA3 region of rats (Gao et al., 2006). The effects of HDAC inhibitors were tested after TBI, including ITF2357, sodium valproate, and sodium butyrate. ITF2357 prevents H3 deacetylation and improves neurobehavioral recovery in mice after TBI (Shein et al., 2009). Post-injury administration of valproate increased H3 and H4 acetylation, decreased blood-brain barrier permeability and improved motor and cognitive function in TBI rats (Dash et al., 2010). Combined with behavioral training, sodium butyrate administration to mice subjected to controlled cortical impact injury improved memory (Dash et al., 2009). Considering that there might be changes in a variety of histone modifications after TBI, we first screened several histone modifications and found that p-H3S10 levels were significantly increased. This increase correlated with the injury severity which depends on the falling weight in our model of TBI (Feeney et al., 1981; Chen et al., 2014). Measurement of p-H3S10 levels at multiple time points showed that p-H3S10 was upregulated at 2 h and on the seventh day after injury but was restored to the control level on the third day after injury. Although secondary injury could start within hours of injury and last up to several weeks, different pathological processes occur and develop within different time frames (Jha et al., 2019). It was reported that cytotoxic edema occurred 1 h after TBI and reached a peak within a few hours (Ito et al., 1996). A study in a closed-skull murine model of TBI showed that the expression levels of aquaporin-4 reached its peak on the seventh day after TBI (Ren et al., 2013). Another study showed that blood-brain barrier destruction might occur twice: within hours and on the fifth day after injury (Jha et al., 2019). It has been further demonstrated that microglia numbers peak at day 7 post-injury then decrease before increasing again at 21 days post-injury (Jin et al., 2012). Therefore, in this study, the increase of p-H3S10 levels at 2 h and on the seventh day after injury may relate to specific pathological processes within different time frames.

Histone phosphorylation is a type of modification that can occur at multiple sites, such as histone H2A serine 10, histone H3 serine 28 and H3S10. Among these modifications, p-H3S10 has been confirmed to participate in mitosis and regulate the activation of corresponding genes (Wei et al., 1999; Nowak and Corces, 2004). The performance of rats in the Morris water maze were found to be positively correlated with the number of p-H3S10-positive dentate gyrus neurons (Carter et al., 2015). The present study provides first evidence for the involvement of ERK-dependent phosphorylation of H3S10 in TBI pathogenesis. The pathophysiological role of histone phosphorylation in TBI remains to be elucidated. However, it has been indicated that post-translational histone modifications including p-H3S10 occur at the promoter region of the CREB (Koshibu et al., 2009). Furthermore, pH3S10 was identified as a permissive post-translational modification in spinal dorsal horn neurons that allows for the ERK-dependent transcription of the immediate early gene (IEG) c-Fos during nociceptive processing (Torres-Pérez et al., 2017). Notably, CREB induces transcription of c-Fos and other IEGs in response to TBI (Dash et al., 1995). Together with the findings of the present study showing an early increase of p-H3S10 in neurons 2 h after TBI, it appears possible that p-H3S10 enhances transcription of c-Fos and other IEGs. This putative role of p-H3S10 may contribute to detrimental processes after TBI. Up-regulation of c-Fos expression has been associated with apoptosis after intracerebral hemorrhage (Chen et al., 2015), kainic-acid induced seizures (Schreiber et al., 1993) and potentiation of c-Fos activity was shown to promote neuronal cell death in a mouse model of Alzheimer’s disease (Choi et al., 2019). Therefore, further studies are required to test the hypothesis that pH3S10 in neurons triggers neuronal cell death by increasing CREB-mediated expression of c-Fos and/or other IEGs in TBI.

Histone H3 serine 10 phosphorylation is regulated by multiple upstream signaling pathways, including the MEK/ERK/MSK pathway and the aurora kinase pathway (Tochiki et al., 2016; Li et al., 2017). When we screened for the upstream kinase of p-H3S10, we found that the p-ERK level was increased.

It has been well studied that ERK signaling controls gene transcription (Yang et al., 2013). ERK signaling pathways play a crucial role in modifications of histone H3 in prothoracicotropic hormone-stimulated prothoracic glands (Gu and Hsieh, 2015). Furthermore, previous work suggested that p-ERK is activated in TBI and participates in the regulation of nuclear factor kappa-B expression (Bhowmick et al., 2019). In our study, the trend of p-ERK level changes with time was synchronized with that of p-H3S10, suggesting that p-ERK regulates p-H3S10 as an upstream kinase of H3S10 in rats. Although AURKB is involved in the regulation of H3S10 phosphorylation in mitosis (Li et al., 2017), in this study, there was no significant change in AURKB levels in brain tissue around the TBI injury site, and no evidence of its regulation of H3S10 phosphorylation was found. Immunofluorescence in brain slices of TBI rats and clinical TBI patients showed that p-H3S10 was mainly expressed in cortical neurons on the injured side and colocalized with p-ERK, which suggested that ERK-H3S10 signaling could be more dominant in neurons than other neural cell types.

It has been reported that U0126, a MEK1/2 inhibitor, can inhibit the formation of p-H3S10 induced by Silver Nanoparticle (Zhao et al., 2019). In addition, U0126 can improve the long-term neurologic function of female rats after experimental stroke (Ahnstedt et al., 2015). To further verify the role of p-ERK/p-H3S10 signaling in TBI, we conducted intracerebroventricular injection of U0126 to inhibit p-ERK and observed the resulting changes in p-H3S10 and consequences on animal behavior in male and female rats. Consistent with our expectations, the level of p-ERK was suppressed, and the upregulation of p-H3S10 in the TBI group was also reversed, which is consistent with the results of previous studies (Chwang et al., 2006). The behavioral evaluation suggested that the injection of U0126 into the lateral ventricle promoted the recovery of motor and cognitive function in TBI rats. The above results verified the activation of p-ERK/p-H3S10 signaling in TBI and provided a potential target for therapeutic intervention in TBI. Although we have not been able to use MEK1/2 inhibitors in humans, p-ERK/p-H3S10 signaling might be a potential target for TBI therapy, and its effects need to be verified in future studies.

Growing evidence suggest that sex could be a biological variable affecting injury outcomes and treatment efficacy after TBI. Overall, human studies report worse outcomes for women than men, while animal studies report better outcomes for females than males (O’Connor et al., 2003; Broshek et al., 2005; Zupan et al., 2018; Gupte et al., 2019). However, multiple factors, including injury severity and experimental injury models, may interact differently with sex to influence TBI results (Gupte et al., 2019; Rubin and Lipton, 2019). In this study, similar neurological and cognitive impairment was shown in male and female TBI rats using a weight drop model, which is consistent with previous findings (Mychasiuk et al., 2016). We further demonstrated that the treatment of U0126 reduced H3S10 phosphorylation and improved motor function recovery and cognitive function in both male and female TBI rats. This result suggests that the phosphorylation-dependent activation of ERK-H3S10 signaling may play an important role in the pathogenesis of TBI in both genders.

Some limitations need to be considered in this study. Western blot analysis for multiple time points was performed only with the minimally required sample size of *n* = 3. Furthermore, we did not examine any relevant changes in p-ERK/p-H3S10 beyond 7 days after TBI. It is also possible that other histone modifications whose levels have not changed within 7 days after TBI are involved in later stages of TBI pathogenesis. Similarly, the neuroprotective effect of U0126 in female rats may differ from that in male rats in the later stages of TBI. Therefore, p-ERK/p-H3S10 signaling might be one of many pathways activated in TBI, and its role in the mechanism of TBI needs to be further investigated and verified in future studies including sex-matched long-term survival cohorts and the implementation of chromatin immunoprecipitation to reveal the specific downstream genes regulated by p-H3S10. Likewise, detailed examinations of human brain tissue specimens are required to gain more insights whether age and sex influences p-ERK/p-H3S10.

## Conclusion

In summary, we found that H3S10 phosphorylation regulated by the ERK pathway is involved in TBI pathogenesis, which may shed new light on the molecular mechanism of TBI and provide new potential interventional targets for TBI treatment.

## Data availability statement

The raw data supporting the conclusions of this article will be made available by the authors, without undue reservation.

## Ethics statement

The studies involving human participants were reviewed and approved by the Ethics Committee of Xiangya Hospital of Central South University. The patients/participants provided their written informed consent to participate in this study. The animal study was reviewed and approved by the Institutional Ethics Committee of Central South University.

## Author contributions

YZh, XY, WZ, ZiD, CB, ZY, and SL carried out the experiments. YZh, XH, and MS participated in the study design and manuscript writing. YZh, ZhD, and YZo analyzed data and presentation. QG participated in the study conception. CH conducted the study design. All authors contributed to manuscript revision, read, and approved the submitted version.

## Footnotes

1
http://www.chictr.org.cn

## Supplementary material

Supplementary Figure S1 Coronal section of the brain showing the correct position of the intracerebroventricular cannula by methylene blue injection 48 h after TBI. The cannula which was fixed to the surface of the skull was removed when taking the brain for sectioning.

Supplementary Figure S2 Measurements of the AURKB protein level at multiple time points showed no significant changes (*n* = 3 rats/group. One-way ANOVA followed by Holm-Sidak’s multiple comparisons test. The values are expressed as the means ± SD).

## Abbreviations

HDAC: histone deacetylase
H3ac: pan-acetylation of histone H3
H3K27ac: acetylation of histone H3 at lysine 27
H3K4me1: monomethylation of histone H3 at lysine 4
H3K9me3: trimethylation of histone H3 at lysine 9
H3K27me3: trimethylation of histone H3 at lysine 27
p-H 3S10: phosphorylation of histone H3 serine 10
p-ERK: phosphorylated form of the extracellular signal-regulated kinase
GCS: Glasgow Coma Scale
ID: inner diameter
OD: outer diameter
MEK: mitogen-activated protein kinase–extracellular signal-regulated kinase kinase
NSS: neurological severity score
AURKB: aurora kinase B
CREB: cAMP response element binding protein
MSK: mitogen and stress-activated protein kinase.
