# Supplementary figures and images for: Extracellular signal-regulated kinase-dependent phosphorylation of histone H3 serine 10 is involved in the pathogenesis of traumatic brain injury
Source: Front Mol Neurosci. 2022 Sep 29;15:828567. doi: 10.3389/fnmol.2022.828567 (PMC9557206; doi:10.3389/fnmol.2022.828567)

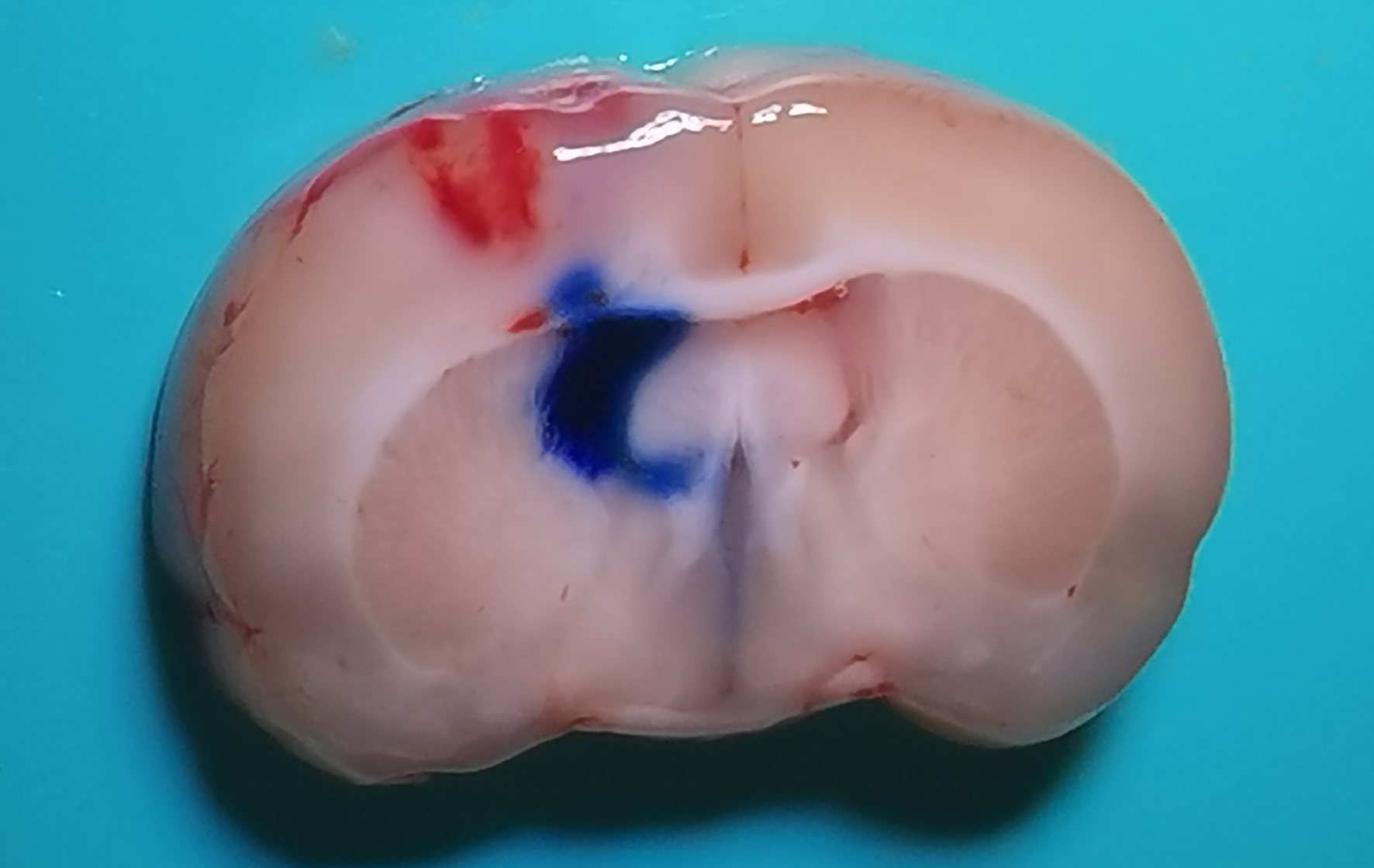

Supplement: Supplementary Figure S1 — Coronal section of the brain showing the correct position of the intracerebroventricular cannula by methylene blue injection 48 h after TBI. The cannula which was fixed to the surface of the skull was removed when taking the brain for sectioning. [file Image_1.TIF]

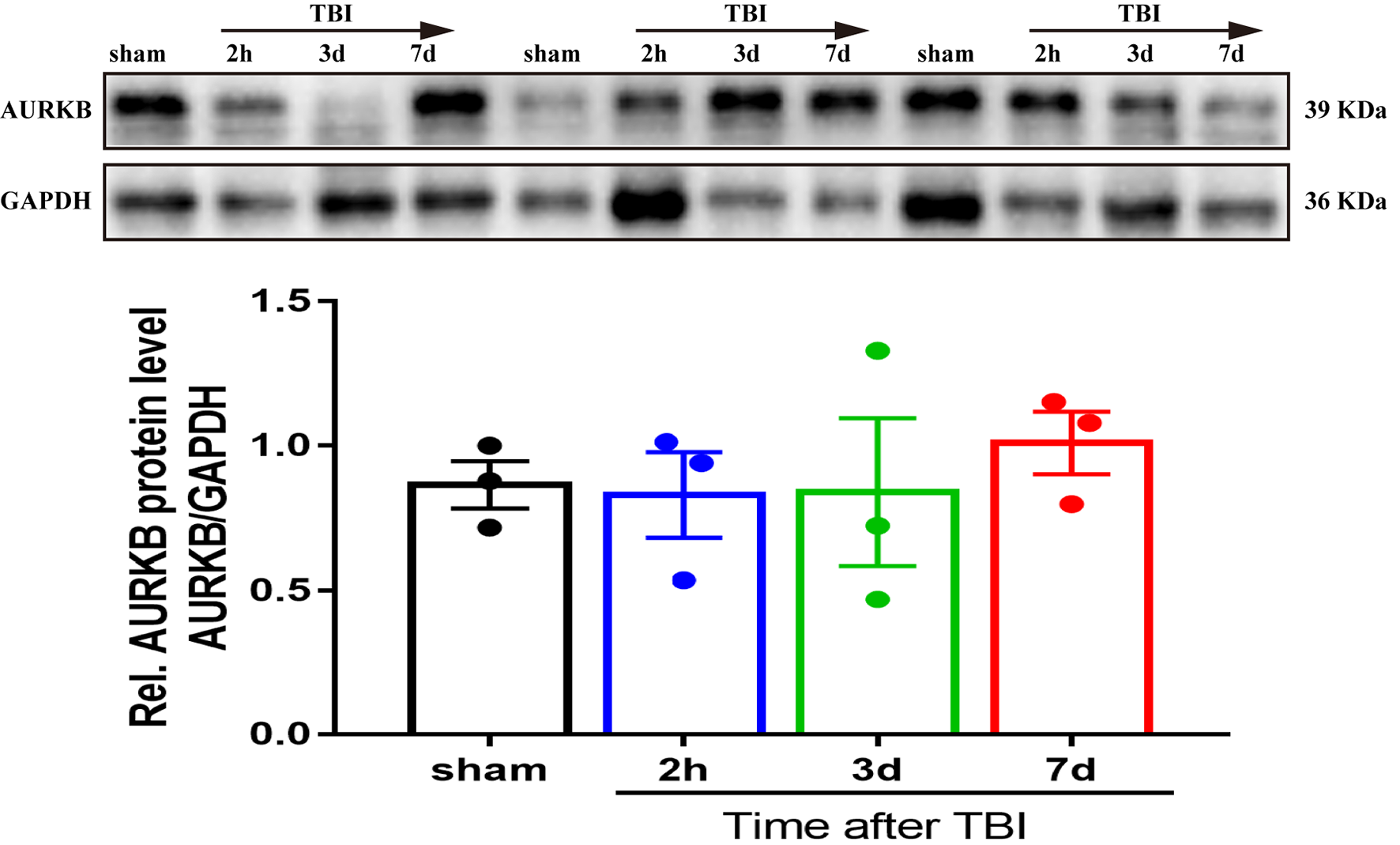

Supplement: Supplementary Figure S2 — Measurements of the AURKB protein level at multiple time points showed no significant changes (n = 3 rats/group. One-way ANOVA followed by Holm-Sidak’s multiple comparisons test. The values are expressed as the means ± SD). [file Image_2.TIF]
